# Effectiveness of Casirivimab-Imdevimab and Sotrovimab During a SARS-CoV-2 Delta Variant Surge: A Cohort Study and Randomized Comparative Effectiveness Trial

**DOI:** 10.1001/jamanetworkopen.2022.20957

**Published:** 2022-07-14

**Authors:** David T. Huang, Erin K. McCreary, J. Ryan Bariola, Tami E. Minnier, Richard J. Wadas, Judith A. Shovel, Debbie Albin, Oscar C. Marroquin, Kevin E. Kip, Kevin Collins, Mark Schmidhofer, Mary Kay Wisniewski, David A. Nace, Colleen Sullivan, Meredith Axe, Russell Meyers, Alexandra Weissman, William Garrard, Octavia M. Peck-Palmer, Alan Wells, Robert D. Bart, Anne Yang, Lindsay R. Berry, Scott Berry, Amy M. Crawford, Anna McGlothlin, Tina Khadem, Kelsey Linstrum, Stephanie K. Montgomery, Daniel Ricketts, Jason N. Kennedy, Caroline J. Pidro, Anna Nakayama, Rachel L. Zapf, Paula L. Kip, Ghady Haidar, Graham M. Snyder, Bryan J. McVerry, Donald M. Yealy, Derek C. Angus, Christopher W. Seymour

**Affiliations:** 1 Clinical Research Investigation and Systems Modeling of Acute Illness (CRISMA) Center, Department of Critical Care Medicine, University of Pittsburgh School of Medicine, Pittsburgh, Pennsylvania; 2 Department of Critical Care Medicine, University of Pittsburgh School of Medicine, Pittsburgh, Pennsylvania; 3 Department of Emergency Medicine, University of Pittsburgh School of Medicine, Pittsburgh, Pennsylvania; 4 Division of Infectious Diseases, Department of Medicine, University of Pittsburgh School of Medicine, Pittsburgh, Pennsylvania; 5 Wolff Center, University of Pittsburgh Medical Center (UPMC), Pittsburgh, Pennsylvania; 6 Supply Chain Management/HC Pharmacy, UPMC, Pittsburgh, Pennsylvania; 7 Clinical Analytics, UPMC, Pittsburgh, Pennsylvania; 8 Division of Cardiology, Department of Medicine, University of Pittsburgh School of Medicine, Pittsburgh, Pennsylvania; 9 Division of Geriatric Medicine, Department of Medicine, University of Pittsburgh School of Medicine, Pittsburgh, Pennsylvania; 10 Health System Office of Healthcare Innovation, UPMC, Pittsburgh, Pennsylvania; 11 Department of Pathology, University of Pittsburgh School of Medicine, Pittsburgh, Pennsylvania; 12 Health Services Division, UPMC, Pittsburgh, Pennsylvania; 13 Department of Medicine, University of Pittsburgh School of Medicine, Pittsburgh, Pennsylvania; 14 Berry Consultants, Austin, Texas; 15 Division of Pulmonary, Allergy, and Critical Care Medicine, Department of Medicine, University of Pittsburgh School of Medicine, Pittsburgh, Pennsylvania

## Abstract

**Importance:**

The effectiveness of monoclonal antibodies (mAbs), casirivimab-imdevimab and sotrovimab, is unknown in patients with mild to moderate COVID-19 caused by the SARS-CoV-2 Delta variant.

**Objective:**

To evaluate the effectiveness of mAb against the Delta variant compared with no mAb treatment and to ascertain the comparative effectiveness of casirivimab-imdevimab and sotrovimab.

**Design, Setting, and Participants:**

This study comprised 2 parallel studies: (1) a propensity score–matched cohort study of mAb treatment vs no mAb treatment and (2) a randomized comparative effectiveness trial of casirivimab-imdevimab and sotrovimab. The cohort consisted of patients who received mAb treatment at the University of Pittsburgh Medical Center outpatient infusion centers and emergency departments from July 14 to September 29, 2021. Participants were patients with a positive SARS-CoV-2 test result who were eligible to receive mAbs according to emergency use authorization criteria.

**Exposure:**

For the trial, patients were randomized to either intravenous casirivimab-imdevimab or sotrovimab according to a system therapeutic interchange policy.

**Main Outcomes and Measures:**

For the cohort study, risk ratio (RR) estimates for the primary outcome of hospitalization or death by 28 days were compared between mAb treatment and no mAb treatment using propensity score–matched models. For the comparative effectiveness trial, the primary outcome was hospital-free days (days alive and free of hospitalization) within 28 days after mAb treatment, where patients who died were assigned −1 day in a bayesian cumulative logistic model adjusted for treatment location, age, sex, and time. Inferiority was defined as a 99% posterior probability of an odds ratio (OR) less than 1. Equivalence was defined as a 95% posterior probability that the OR was within a given bound.

**Results:**

A total of 3069 patients (1023 received mAb treatment: mean [SD] age, 53.2 [16.4] years; 569 women [56%]; 2046 had no mAb treatment: mean [SD] age, 52.8 [19.5] years; 1157 women [57%]) were included in the prospective cohort study, and 3558 patients (mean [SD] age, 54 [18] years; 1919 women [54%]) were included in the randomized comparative effectiveness trial. In propensity score–matched models, mAb treatment was associated with reduced risk of hospitalization or death (RR, 0.40; 95% CI, 0.28-0.57) compared with no treatment. Both casirivimab-imdevimab (RR, 0.31; 95% CI, 0.20-0.50) and sotrovimab (RR, 0.60; 95% CI, 0.37-1.00) were associated with reduced hospitalization or death compared with no mAb treatment. In the clinical trial, 2454 patients were randomized to receive casirivimab-imdevimab and 1104 patients were randomized to receive sotrovimab. The median (IQR) hospital-free days were 28 (28-28) for both mAb treatments, the 28-day mortality rate was less than 1% (n = 12) for casirivimab-imdevimab and less than 1% (n = 7) for sotrovimab, and the hospitalization rate by day 28 was 12% (n = 291) for casirivimab-imdevimab and 13% (n = 140) for sotrovimab. Compared with patients who received casirivimab-imdevimab, those who received sotrovimab had a median adjusted OR for hospital-free days of 0.88 (95% credible interval, 0.70-1.11). This OR yielded 86% probability of inferiority for sotrovimab vs casirivimab-imdevimab and 79% probability of equivalence.

**Conclusions and Relevance:**

In this propensity score–matched cohort study and randomized comparative effectiveness trial, the effectiveness of casirivimab-imdevimab and sotrovimab against the Delta variant was similar, although the prespecified criteria for statistical inferiority or equivalence were not met. Both mAb treatments were associated with a reduced risk of hospitalization or death in nonhospitalized patients with mild to moderate COVID-19 caused by the Delta variant.

**Trial Registration:**

ClinicalTrials.gov Identifier: NCT04790786

## Key Points

**Question:**

Is monoclonal antibody (mAb) treatment effective in nonhospitalized patients with COVID-19 caused by the Delta variant?

**Findings:**

In this propensity score–matched cohort study (n = 3069) and randomized comparative effectiveness trial (n = 3558), mAb treatment (casirivimab-imdevimab or sotrovimab) compared with no mAb treatment was associated with reduced hospitalization or death by 28 days, and sotrovimab compared with casirivimab-imdevimab resulted in 86% probability of inferiority and 79% probability of equivalence in the odds of improvement in hospital-free days within 28 days after mAb treatment.

**Meaning:**

Findings of this study suggest that casirivimab-imdevimab and sotrovimab were both associated with reduced risk of hospitalization or death and had similar effectiveness, although they did not meet the prespecified criteria for statistical inferiority or equivalence.

## Teaser

This combined cohort study and randomized comparative effectiveness trial examines the hospitalization and mortality outcomes among outpatients receiving monoclonal antibodies to treat COVID-19 caused by the SARS-CoV-2 Delta variant.

## Introduction

Monoclonal antibodies (mAbs) were granted emergency use authorization (EUA) by the US Food and Drug Administration for treatment of mild to moderate COVID-19.^1,2^ However, their effectiveness against the Delta variant of SARS-CoV-2 is unknown.

In February 2021, University of Pittsburgh Medical Center (UPMC) partnered with the US COVID-19 Response Team to expand the clinical use and evaluate the effectiveness of bamlanivimab, bamlanivimab and etesevimab, and casirivimab-imdevimab using a learning health system approach. This approach embeds knowledge generation into daily practice to seek continuous improvement in care.^3,4,5^ In April 2021, the EUA for bamlanivimab was revoked. In June 2021, UPMC partnered with GlaxoSmithKline and Vir Biotechnology to make sotrovimab available to eligible patients eligible per the EUA to receive sotrovimab and to evaluate its effectiveness. Because the US government paused the distribution of bamlanivimab and etesevimab and the Delta variant became the dominant SARS-CoV-2 strain in the US in summer 2021, we evaluated the effectiveness of casirivimab-imdevimab and sotrovimab.^6,7^

In this study, we evaluated casirivimab-imdevimab and sotrovimab from July 14 to September 29, 2021, and released the results because of the Delta variant public health crisis.^8^ We aimed to evaluate the effectiveness of mAb against the Delta variant compared with no mAb treatment and to ascertain the comparative effectiveness between mAb treatments.

## Methods

This study comprised (1) a propensity score–matched cohort study of mAb treatment vs no mAb treatment and (2) a randomized comparative effectiveness trial of casirivimab-imdevimab and sotrovimab, which involved additional data collection and analysis of the trial results. The UPMC Quality Improvement Committee approved the study, and the University of Pittsburgh Institutional Review Board approved the additional data collection and analyses. Patients provided verbal consent for their participation. We followed the Consolidated Standards of Reporting Trials (CONSORT) reporting guideline, and reported methods and results in accordance with the CONSORT Extension for Pragmatic Trials checklist.^4,16^

### Study Setting, Data Sources, and Exposure

The UPMC is a 40-hospital integrated health system principally located in central and western Pennsylvania. After an EUA was granted for bamlanivimab on November 9, 2020, UPMC developed a mAb treatment infrastructure.^9,10,11^ On November 21, 2020, the UPMC Pharmacy and Therapeutics Committee wrote a therapeutic interchange policy in response to the issuance of an EUA for casirivimab-imdevimab and unpredictable mAb supply. The policy considered all available mAb treatments equivalent; a patient could receive any mAb on the basis of local inventory. The policy was updated on July 9, 2021, to include sotrovimab. All pharmacies that supplied to all infusion sites had equal opportunity to order any available mAb from a central supply facility. Prescribers were required to provide and review with the patient EUA mAb fact sheets at the time of referral and to explain that the patient could receive any EUA-governed mAb under the therapeutic interchange policy. Patients eligible per the EUA to receive mAb treatment were referred using the UPMC electronic medical record for UPMC prescribers or paper order for non-UPMC prescribers. A centralized team of nurses, administrators, pharmacists, and physicians reviewed orders daily to confirm criteria for receipt. Decentralized nursing teams then contacted and scheduled eligible patients for infusions.

We used the electronic medical record to access key clinical data, including detailed sociodemographic characteristics (eg, race and ethnicity, which were self-reported by patients and included the categories of Black, White, other [including Alaska Native, American Indian, Asian, Filipino, Indian, Native Hawaiian, and Pacific Islander], and unknown), medical history, and hospitalization after mAb treatment, which we augmented by performing manual review and data collection. We linked the deidentified primary data sources using common variables within the UPMC data systems that are aggregated in its clinical data warehouse. Adverse events were reported according to federal safety and reporting guidelines, as required by the EUAs. We queried the Social Security Administration Death Master File for vital status.^12^

Both casirivimab-imdevimab and sotrovimab were provided as specified by their EUAs and administered intravenously to all participants in this study. Patients who received casirivimab-imdevimab via subcutaneous injection were excluded from the analysis. Some patients in the present study participated as control patients in a previous study comparing subcutaneous with intravenous administration of casirivimab-imdevimab.^13^

### Propensity Score–Matched Cohort Study 

The propensity score–matched cohort study population was derived from patients who received mAb treatment at UPMC outpatient infusion centers from July 14 to September 29, 2021. We derived a comparator group from the same at-risk population by identifying nonhospitalized patients with a positive polymerase chain reaction or antigen test result for SARS-CoV-2 during the same period who were eligible for but did not receive mAb treatment.

For patients who received mAb treatment, the follow-up period began on the day of treatment. For patients with no mAb treatment (comparator group), the follow-up period began 1 day after their positive SARS-CoV-2 test result date, which corresponded to the shortest duration from test positivity to mAb treatment for patients who received mAb treatment. We restricted this analysis to patients who received mAb treatment at UPMC infusion centers because no comparator group was present for patients who were treated in UPMC emergency departments (EDs).

### Randomized Comparative Effectiveness Trial 

This randomized comparative effectiveness trial used a bayesian adaptive platform and provided the mAb therapeutic interchange via randomization. The UPMC Quality Improvement Committee approved randomization to a specific mAb treatment under the therapeutic interchange policy, and the University of Pittsburgh Institutional Review Board considered the therapeutic interchange system to be quality improvement.

A custom application built into the electronic medical record linked local inventory to patient encounters and provided randomized mAb allocation at the time of mAb referral. With this application, randomization was triggered when clinicians entered into the electronic medical record a generic mAb referral order. When the patient's medical record number was entered and the randomize button was clicked (by a centralized mAb operations team member at infusion centers and by a pharmacist at EDs), a random mAb was generated. The patient's randomized treatment was automatically recorded in the data and analytics team database.

Patients received mAb therapy as part of routine care within the EUA. The prescriber and/or patient could request a specific mAb, and such a request was granted if the desired mAb was available. With a pragmatic adaptive design, this trial had no set sample size; the sample size was dependent on case volume, mAb treatment capacity, and meeting predetermined statistical thresholds for equivalence or inferiority. Details of the trial methods and implementation have been reported, and the trial protocol is provided in Supplement 1.^14,15^

### Patient Cohort

**Figure 1.**
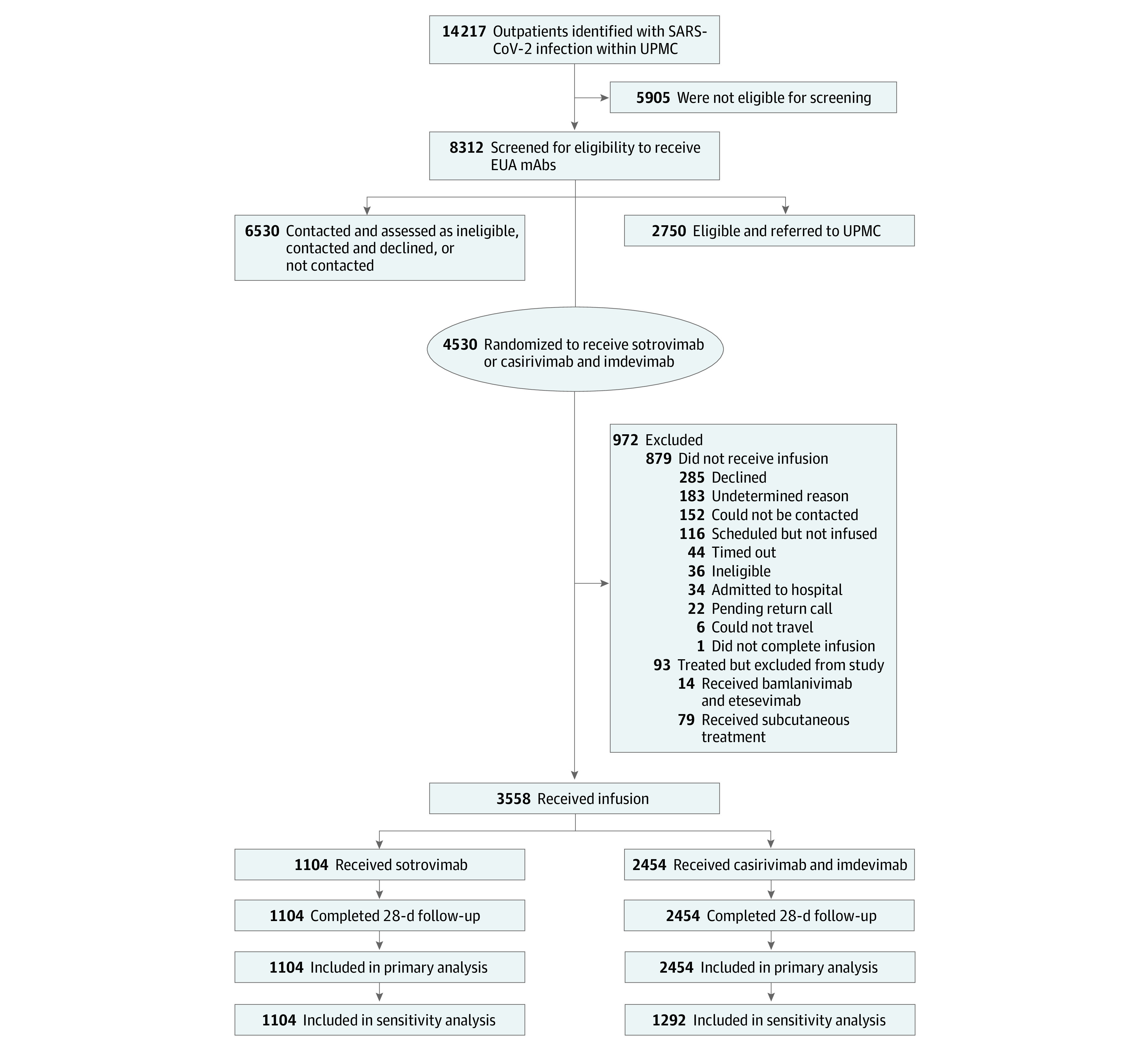
Flow Diagram of Study Participants Because of pharmacy logistics, 6 patients who received sotrovimab were randomized to receive casirivimab-imdevimab in a University of Pittsburgh Medical Center (UPMC) location where both monoclonal antibodies (mAbs) were available, and 16 patients who received casirivimab-imdevimab were randomized to receive sotrovimab in a UPMC location where both mAbs were available. Because of mAb shortages, 1162 patients received treatment in a location where only casirivimab-imdevimab was available at the time of treatment. EUA indicates emergency use authorization.

The primary analysis population consisted of patients who were randomized to receive sotrovimab or casirivimab-imdevimab and who received infusion or mAb treatment at UPMC sites during the study period (Figure 1). Given that all treatment groups included mAbs, there was no anticipated association between lack of infusion and the randomized treatment. Because of episodic mAb shortages, some patients received mAb treatment at sites with only 1 available mAb at the time of treatment. We included these patients in the primary analysis because both prescribing physicians and patients were blinded to drug availability at the time of randomization and patients could receive mAb treatment at a site that was different from the randomization site, pending scheduling availability.

### Outcomes

For the propensity score–matched cohort study, the primary outcome was hospitalization or death by 28 days. Secondary outcomes included the 28-day hospitalization rate and 28-day mortality rate.

For the randomized comparative effectiveness trial, the primary outcome was hospital-free days up to day 28 after mAb treatment. This outcome was an ordinal end point, with death as the worst outcome (labeled as −1) followed by the length of time alive and free of hospitalization, such that the best outcome would be 28 hospital-free days. If a patient had intervening days free of hospitalization and was then rehospitalized, the patient was given credit for the intervening days as free of hospitalization. We used a different primary outcome for the comparative effectiveness trial vs the cohort study because we hypothesized that, in addition to potential differences in 28-day hospitalization and mortality rates between mAb types (sotrovimab, casirivimab-imdevimab, and casirivimab-imdevimab or sotrovimab), there may be differences in hospital length of stay. The ordinal primary outcome for the trial allowed a single summary measure to encompass these potential differences.

The secondary outcome for the trial was mortality at 28 days. We evaluated outcomes stratified by infusion location (ED vs infusion center) and the frequency of adverse events. We assessed the prevalence of SARS-CoV-2 variants in a random subset of enrolled patients and in the Pennsylvania catchment area using GISAID (Global Initiative on Sharing All Influenza Data)^7^ (eFigure 1 in Supplement 2).

### Statistical Analysis

For the propensity score–matched cohort study, we performed 3 analyses. We used propensity score matching to compare patients who were eligible but received no mAb treatment with patients who received (1) any mAb (casirivimab-imdevimab or sotrovimab), (2) casirivimab-imdevimab, and (3) sotrovimab. Matching the patients who received mAb treatment with control patients with no mAb treatment required full covariate data and a suitable match. Propensity scores were derived from multivariable logistic regression models that were fit from variables, with mAb treatment as the response variable, and forward stepwise selection of pretreatment explanatory variables at *P* < .15. We included variables that were deemed biologically relevant (eg, age) before stepwise selection (eTable 1 in Supplement 2). We used a 1:2 matching ratio (mAb treatment or no mAb treatment) with a maximum propensity score probability difference of 0.01. We compared the characteristics of patients who received mAb treatment vs patients with no mAb treatment using unpaired, 2-tailed *t* tests for continuous variables and χ ^2^ tests for categorical variables before and after matching by propensity score. The primary outcome analysis consisted of the 1:2 matched comparisons of patients who received mAb treatment and patients with no mAb treatment, with the study outcomes expressed as an effect estimate of treatment (risk ratio [RR] with 95% CI). Because of the similarity of patients who received casirivimab-imdevimab and those who received sotrovimab and to ensure the same reference group (patients with no mAb treatment) for comparison, we used the full set of matched control patients in all analyses. In a sensitivity analysis, we also conducted unmatched analyses, including adjustment by propensity score and inverse probability weighting.

For the comparative effectiveness trial, the statistical analysis plan was written by blinded investigators (D.T.H., C.W.S., D.C.A., S.B.) before data lock and analysis. The platform was designed to continuously evaluate multiple mAb treatments, with randomization continuing until the predetermined statistical thresholds were met. The trial launched with equal randomization and planned interim analyses for adaptive randomization, where the mAb treatment that performed better would be given higher randomization probabilities. The mAb group at the first adaptive analysis with the largest sample size was specified as the referent group because there was no non-mAb control group, and all patients received active treatment. An unblinded statistical analysis committee conducted interim and final analyses using the RStan package, version 2.21.0, in R, version 4.0.5 (R Foundation for Statistical Computing). The committee reported the results to the UPMC chief medical officer (D.M.Y.), who functioned as a data and safety monitor for the present study.

The primary model was a bayesian cumulative logistic model that adjusted for treatment location (infusion center or ED), age (<30, 30-39, 40-49, 50-59, 60-69, 70-79, and ≥80 years), sex, and time (2-week periods). The comparison between individual mAb treatments was based on the relative odds ratio (OR) between 2 groups for the ordinal primary outcome. An OR for a group to a comparator that was greater than 1 implied improved outcomes on the ordinal scale. A sliding scale with different levels of equivalence bounds was predefined (Statistical Analysis Plan and Study Protocol in Supplement 1). Equivalence between 2 groups was defined as a 95% posterior probability that the OR was within a given OR bound. Inferiority of 1 group vs another group was defined as a 99% posterior probability of an OR less than 1.

We conducted a sensitivity analysis that excluded patients who received mAb treatment at a site with only 1 available mAb treatment option. We conducted a priori subgroup analyses by vaccination status (fully vaccinated, partially vaccinated, unvaccinated, or unknown), symptom onset (>5 days or ≤5 days), and location (infusion center or ED). Because of the SARS-CoV-2 Delta variant public health crisis, we unblinded and analyzed patients who were randomized through September 29, 2021, before having knowingly met any prespecified statistical threshold.

## Results

In the propensity score–matched cohort study, 3069 patients were included. In the unmatched group, 1028 patients received mAb treatment and had full covariate data for matching vs 5171 patients with no mAb treatment. Patients who received mAb treatment were less likely than patients with no mAb treatment to be of Black race, had lower mean Charlson Comorbidity Index scores, and were more likely to have greater body mass index (calculated as weight in kilograms divided by height in meters squared). After propensity score matching, we found that patients who received mAb treatment and those with no mAb treatment were similar for all variables included in the propensity score model, the distribution of propensity scores, and variables that were excluded from the model (eFigure 2 and eTable 1 in Supplement 2).

In the propensity score–matched cohort, of the 1023 patients who received mAb treatment at an infusion center (mean [SD] age, 53.2 [16.4] years; 569 women [56%] and 454 men [44%]), 35 (3%) were hospitalized or died. Of the 2046 patients with no mAb treatment (mean [SD] age, 52.8 [19.5] years; 1157 women [57%] and 889 men [43%]), 174 (9%) were hospitalized or died (Table 1). The mortality rate was less than 1% (1 of 1023) in patients who received mAb treatment and 3% (60 of 2046) in patients with no mAb treatment. In the propensity score–matched analysis (Table 1), patients who received mAb treatment had lower adjusted risk of hospitalization or death (RR, 0.40; 95% CI, 0.28-0.57) compared with patients with no mAb treatment. Among the 712 patients who received casirivimab-imdevimab, 19 (3%) were hospitalized and 1 died (<1%), with lower adjusted risk of hospitalization or death (RR, 0.31; 95% CI, 0.20-0.50) compared with patients with no mAb treatment. Among the 311 patients who received sotrovimab, 16 (5%) were hospitalized and none died, yielding lower adjusted risk of hospitalization or death (RR, 0.60; 95% CI, 0.37-1.00) compared with patients with no mAb treatment. Unmatched analyses had similar results (eTable 2 in Supplement 2).

**Table 1.** Adjusted Hospitalization and Mortality Rates in Patients Who Received mAb Treatment vs No mAb Treatment

	No. of events		28-d Event rate, %		RR estimates	
	Received treatment	No treatment	Received treatment	No treatment	RR (95% CI)	*P* value
**Casirivimab-imdevimab **						
No. of patients	712	2046	NA	NA	NA	NA
Hospitalization or mortality	19	174	3	9	0.31 (0.20-0.50)	<.001
Hospitalization	19	134	3	7	0.41 (0.25-0.65)	<.001
Mortality	1	60	<1	3	0.05 (0.01-0.34)	.003
**Sotrovimab **						
No. of patients	311	2046	NA	NA	NA	NA
Hospitalization or mortality	16	174	5	9	0.60 (0.37-1.00)	.05
Hospitalization	16	134	5	7	0.79 (0.47-1.30)	.35
Mortality	0	60	0	3	0	
**Any mAb (casirivimab-imdevimab or sotrovimab)**						
No. of patients^a^	1023	2046	NA	NA	NA	NA
Hospitalization or mortality	35	174	3	9	0.40 (0.28-0.57)	<.001
Hospitalization	35	134	3	7	0.52 (0.36-0.75)	<.001
Mortality	1	60	<1	3	0.03 (0.01-0.34)	<.001

Abbreviations: mAb, monoclonal antibody; NA, not applicable; RR, risk ratio.

^a^ Patients who received mAb treatment (n = 1023) were part of the propensity score–matched cohort who received infusion at a University of Pittsburgh Medical Center site, out of the total cohort of patients who received mAb treatment from July 14 to September 29, 2021 (n = 3558). Patients with no mAb treatment (n = 2046) were part of the 1:2 propensity score–matched comparator group of nonhospitalized patients with a positive SARS-CoV-2 test result during the same period who were eligible for but did not receive mAb treatment.

In the randomized comparative effectiveness trial, among 4530 patients who were referred and randomized, 3558 (79%) received infusion (2454 were randomized to casirivimab-imdevimab, and 1104 were randomized to sotrovimab) (Figure 1). The mean (SD) age across the treatment groups was 54 (18) years, and the cohort included 1919 women (54%) and 1639 men (46%), 84% (n = 1919) of whom had White race and ethnicity (Table 2). Baseline characteristics were similar across groups (Table 2). Thirty-two patients (1%) were aged 12 to 17 years.

**Table 2.** Characteristics of Patients Who Received mAb Treatment

	No. (%)		
	Casirivimab- imdevimab	Sotrovimab	Entire cohort
No. of patients^a^	2454 (69)	1104 (31)	3558
Patient characteristics			
Age, mean (SD), y	54 (18)	53 (18)	54 (18)
Sex			
Female	1320 (54)	599 (54)	1919 (54)
Male	1134 (46)	505 (46)	1639 (46)
Race and ethnicity^b^			
Black	252 (10)	161 (15)	413 (12)
White	2089 (85)	892 (81)	2981 (84)
Other^c^	50 (2)	27 (2)	77 (2)
Unknown	63 (3)	24 (2)	87 (2)
Vaccination status			
Fully vaccinated	632 (26)	300 (27)	932 (26)
Partially vaccinated	82 (3)	39 (3.5)	121 (4)
Unvaccinated	499 (20)	182 (17)	681 (19)
Unknown	1241 (51)	583 (53)	1824 (51)
BMI, mean (SD)	32.7 (8.0)	32.4 (7.7)	32.6 (7.9)
Timing of randomization, mean (SD)			
Days from randomization to infusion	0.7 (1.2)	0.6 (1.0)	0.7 (1.2)
Days from symptoms to randomization	4.5 (2.0)	4.6 (2.1)	4.5 (2.0)
Days from symptoms to infusion	6.0 (1.9)	5.8 (2.0)	5.9 (1.9)
**mAb treatment sites**			
Location			
Infusion center	1055 (43)	456 (41)	1511 (43)
Emergency department	1399 (57)	648 (59)	2047 (58)
**mAb treatment eligibility**			
Qualifying EUA criteria			
Age ≥65 y	772 (32)	311 (28)	1083 (30)
Age 12-17 y with qualifying criteria^d^	26 (1)	6 (1)	32 (1)
BMI >25	1453 (59)	639 (58)	2092 (59)
CKD	120 (5)	46 (4)	166 (5)
Diabetes	281 (11)	153 (14)	434 (12)
Down syndrome	1 (0)	2 (0)	3 (0)
Current or former smoker	359 (15)	178 (16)	537 (15)
Current or history of SUD	26 (1)	9 (1)	35 (1)
Immunosuppressive disease or treatment^e^	383 (16)	169 (15)	552 (16)
Sickle cell disease	4 (0)	0 (0)	4 (0)
CVD	378 (15)	181 (16)	559 (16)
Hypertension	732 (30)	341 (31)	1073 (30)
Respiratory condition	536 (22)	243 (22)	779 (22)

Abbreviations: BMI, body mass index (calculated as weight in kilograms divided by height in meters squared); CKD, chronic kidney disease; CVD, cardiovascular disease; EUA, emergency use authorization; mAb, monoclonal antibody; SUD, substance use disorder.

^a^ These patients represent all patients who received intravenous casirivimab-imdevimab and sotrovimab from July 14 to September 29, 2021 (n = 3558).

^b^ Race and ethnicity were self-reported by patients.

^c^ Other race and ethnicity included Alaska Native, American Indian, Asian, Filipino, Indian, Native Hawaiian, and Pacific Islander.

^d^ As per the EUA, qualifying criteria for pediatric patients were the same as for adults and included diabetes, elevated BMI, and chronic kidney disease.

^e^ Immunosuppressive disease or treatment was defined as a history of HIV, cancer, transplant (solid organ, stem cell, or bone marrow), chemotherapy treatment, lupus, rheumatoid arthritis, or liver disease.

The most common risk factors were body mass index higher than 25, age 65 years or older, and hypertension. The mean (SD) duration of symptom onset to infusion was 5.9 (1.9) days. Of 972 patients who were randomized but excluded from analysis, 437 (45%) declined treatment or could not be contacted, 183 (19%) had undetermined reasons, and 116 (12%) were scheduled but did not receive infusion (Figure 1). One patient requested a specific mAb that was different from the randomized mAb and was analyzed as having received infusion. All patients (n = 79) who were tested for SARS-CoV-2 variant type had the Delta variant, consistent with Pennsylvania GISAID data for this period that showed nearly all variants in the state were Delta (eFigure 1 in Supplement 2).

### Primary and Secondary Outcomes

**Figure 2.**
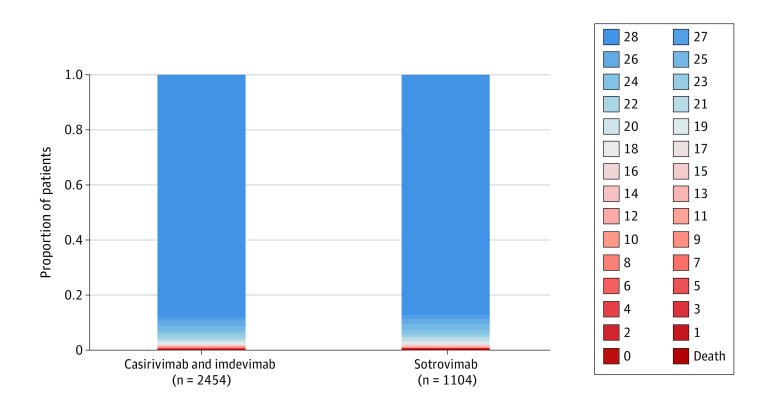
Hospital-Free Days to Day 28 Primary outcome is displayed as horizontally stacked proportions of patients by monoclonal antibody type. Red represents worse values, and blue represents better values. The median adjusted odds ratio (OR) from the primary analysis, using a bayesian cumulative logistic model, was 0.88 (95% credible interval, 0.70-1.11) for sotrovimab vs casirivimab-imdevimab. This OR yielded 86% probability of inferiority for sotrovimab vs casirivimab-imdevimab and a 79% probability of equivalence between the 2 mAbs at the first prespecified bound.

The median (IQR) hospital-free days were 28 (28-28) (Table 3, Figure 2). Compared with patients who received casirivimab-imdevimab, those who received sotrovimab had a posterior median adjusted OR in the primary model of 0.88 (95% credible interval, 0.70-1.11). This OR resulted in an 86% probability of inferiority for sotrovimab vs casirivimab-imdevimab. The probability of equivalence between sotrovimab and casirivimab-imdevimab at the first prespecified bound was 79%. No comparison met the prespecified criteria for statistical inferiority or equivalence.

**Table 3.** Primary Outcomes and Analysis of Randomized Comparative Effectiveness Trial

	Casirivimab-imdevimab	Sotrovimab
No. of patients^a^	2454	1104
**Primary outcome**		
Hospital-free days, median (IQR)	28 (28-28)	28 (28-28)
Patients with 28 hospital-free days, No. (%)	2163 (88)	964 (87)
Subcomponents of hospital-free days		
Deaths, No. (%)	12 (1)	7 (1)
Hospital length of stay among hospitalized patients, median (IQR), d	4 (2-8)	4 (2-8)
Hospitalization, No. (%)	291 (12)	140 (13)
After mAb infusion		
In emergency department	262/1399 (19)	113/648 (17)
In infusion center	29/1055 (3)	27/456 (6)
**Primary analysis**		
Adjusted OR		
Mean (SD)	0.89 (0.11)	1 [Reference]
Median (95% credible interval)	0.88 (0.70-1.11)	1 [Reference]
Probability of inferiority, %		
To casirivimab-imdevimab	NA	86
To sotrovimab	14	NA
Probability of equivalence within the following OR bound, %		
0.25	79	79
0.20	67	67
0.15	53	53
0.10	36	36
0.05	19	19

Abbreviations: mAb, monoclonal antibody; NA, not applicable; OR, odds ratio.

^a^ These patients represent all patients who received intravenous casirivimab-imdevimab and sotrovimab from July 14 to September 29, 2021 (n = 3558).

For patients who received casirivimab-imdevimab (n = 2454), the 28-day mortality rate was less than 1% (n = 12) and hospitalization rate was 12% (n = 291). For patients who received sotrovimab (n = 1104), the 28-day mortality rate was less than 1% (n = 7) and hospitalization rate was 13% (n = 140) (Table 3). For patients who received mAb treatment in an ED, rates of hospitalization after treatment were 19% (262 of 1399) for casirivimab-imdevimab and 17% (113 of 648) for sotrovimab. For patients who received mAb treatment in an infusion center, rates of hospitalization after treatment were 3% (29 of 1055) for casirivimab-imdevimab and 6% (27 of 456) for sotrovimab (Table 3).

### Sensitivity and Subgroup Analyses

We performed multiple sensitivity analyses to determine the robustness of the results. First, we excluded 1162 patients who received casirivimab-imdevimab at a location where only casirivimab-imdevimab was available and found similar results (81% probability of sotrovimab inferiority to casirivimab-imdevimab, and 79% equivalence). No subgroup analysis except location met the prespecified thresholds for statistical inferiority or equivalence. Among the 1511 patients who received mAb treatment in an infusion center, the median OR of sotrovimab compared with casirivimab-imdevimab was 0.48 (95% credible interval, 0.28-0.84), resulting in a 99.5% probability of inferiority (eTable 3 in Supplement 2).

### Adverse Events

Among the 2454 patients who received casirivimab-imdevimab, 17 (<1%) reported adverse events and 7 (<1%) reported severe adverse events, making them rare. Similarly, for the 1104 patients who received sotrovimab, 6 (<1%) reported adverse events and 4 (<1%) reported severe adverse events. The most commonly reported adverse events were flushing, itching, breathing difficulties, and chest tightness or pain.

## Discussion

During the Delta variant surge, we found that casirivimab-imdevimab and sotrovimab were each associated with a reduced risk-adjusted hospitalization and death among patients with mild to moderate COVID-19 compared with no mAb treatment. In a randomized comparative effectiveness trial, no primary analysis met a prespecified trigger for conclusions of inferiority or equivalence, although the subgroup analysis of patients who received mAb treatment in an infusion center showed superiority of casirivimab-imdevimab over sotrovimab.

The Delta variant of SARS-CoV-2 was observed in late 2020 and became the dominant strain worldwide by July 2021. Yet, early evidence of the efficacy of mAb was reported before the emergence of the Delta variant. The present study extended previous work^5^ and found that mAbs were associated with improved outcomes in patients with the Delta variant in a cohort with a robust sample size and methods to adjust for treatment selection and confounding. Future work is needed to ascertain the effectiveness of mAbs compared with recently authorized oral antivirals against the Omicron variant of SARS-CoV-2.

We found no difference when comparing the effectiveness of mAb according to predefined statistical thresholds. This finding could be associated with the true absence of a difference or the insufficient power to detect a difference owing to our decision to evaluate the trial results early because of pandemic emergency. In an a priori subgroup, we observed a 99.5% probability of inferiority in patients who received mAb treatment at an infusion center. Although not a primary analysis result, this finding, if replicated, suggests that casirivimab-imdevimab may be preferred at least for patients with the Delta variant who received mAb treatment at an infusion center. Patients who present to an ED may have a greater illness severity than those who present for a scheduled appointment at an infusion center, and their illness severity may be the main determinant of their subsequent course, not the mAb type received.

### Limitations

This study has several limitations. First, the cohort study of mAb treatment vs no mAb treatment may be subject to residual or unmeasured confounding. Second, we evaluated the results of the comparative effectiveness trial early because of the Delta variant crisis; thus, the primary analysis was inconclusive. Third, this study was restricted to the patients in the catchment area of UPMC, limiting generalizability. Fourth, the speed with which new SARS-CoV-2 variants emerge can overtake analyses of COVID-19 investigations, decreasing their clinical relevance. Ideally, each mAb therapy should be evaluated against new variants, given the clinical and laboratory evidence of varying effectiveness by variant type.^17,18,19^ Fifth, we did not capture time to negative nasopharyngeal swab. Sixth, the study was not powered to identify treatment effects by symptom onset or vaccination status.

## Conclusions

In nonhospitalized patients with mild to moderate COVID-19 caused by the Delta variant, casirivimab-imdevimab and sotrovimab were both associated with reduced risk of hospitalization or death. The effectiveness of these mAb treatments against the Delta variant appeared to be similar, although prespecified criteria for statistical inferiority or equivalence were not met.

## Notes

Supplement 1.
Statistical Analysis Plan and Study Protocol

Supplement 2. **eFigure 1.** SARS-CoV-2 Variants of Concern Proportion in Pennsylvania During the Study **eFigure 2.** Propensity Scores for Unmatched and Matched Patients **eTable 1.** Comparison of Characteristics of Unmatched and Propensity Matched Patients **eTable 2.** Primary and Secondary Outcomes in an Unmatched Cohort of Patients Receiving Monoclonal Antibody Treatment and an At-Risk Population of Patients Not Receiving Monoclonal Antibody Treatment **eTable 3.** Subgroup Analyses of the Randomized Comparative Effectiveness Trial, Comparing Sotrovimab to Casirivimab and Imdevimab

Supplement 3.
Data Sharing Statement
