# Supplementary material for: Effectiveness of Casirivimab-Imdevimab and Sotrovimab During a SARS-CoV-2 Delta Variant Surge: A Cohort Study and Randomized Comparative Effectiveness Trial
Source: JAMA Netw Open. 2022 Jul 14;5(7):e2220957. doi: 10.1001/jamanetworkopen.2022.20957 (PMC10881222; doi:10.1001/jamanetworkopen.2022.20957)
Supplement: Supplement 1. — Statistical Analysis Plan and Study Protocol [file jamanetwopen-e2220957-s001.pdf]

Statistical Analysis Plan

Huang DT, McCreary EK, Bariola JR, et al. Effectiveness of casirivimab and imdevimab, and sotrovimab, during a Delta variant surge: a prospective cohort study and comparative effectiveness randomized trial

Final Version 1.1, July 26, 2021

Design for UPMC Antibody Treatment and Evaluation Center

by Berry Consultants

Summary of Changes

Page #	Section	Reason for Change
Amendment 1 - July 26, 2021		
3	Model Convergence	Clarify handling of model convergence.
6	Modeling Treatment Heterogeneity	Additional use of Pennsylvania statewide variant data as a surrogate for patient-level variant data
November 4, 2021		
7	Appendix Read-out	Provided details of the final analytical read-out.

Trial Design Introduction

This trial is a platform trial investigating the relative safety and efficacy of multiple monoclonal antibody (mAB) regimens for the treatment of COVID-19 illness. This document describes the statistical details for the trial investigating the relative efficacy of multiple mAB regimens for patients meeting the FDA emergency use authorization (EUA).

The trial randomly allocates which mAB regimen patients receive and will evaluate their comparative effectiveness. Adaptive randomization will be utilized where mAB arms that are performing better will be given higher randomization probabilities. Different mAB arms may be added during the course of the trial and different mAB strategies may be dropped for futility. For the primary analysis of patients within the FDA EUA, there will be no control arm and all arms will be directly compared to all other arms for relative efficacy.

Treatment Arms

The trial may investigate multiple mAB strategy arms. Let k the number of active mAB strategies at any time in the trial. We label these arms as $a=1,\ldots,k$.

Primary Endpoint

The primary endpoint in the trial is hospital-free days (HFDs). The endpoint is a composite of death and number of days alive and free of the hospital. The worst outcome is that a patient dies within 28 days. This outcome is labeled as a –1 HFDs. For patients who do not die within 28 days the primary endpoint is the number of days alive and free of hospitalization. For patients alive at day 28 the endpoint is characterized as an integer value with the number of days free of hospital admission, with possible

values 0, 1, 2, 3, …, 28. For statistical analyses the endpoint is modeled as an ordered categorical variable. If a patient has intervening days free of hospital and then has a re-hospitalization the patient will be given credit for the intervening days as “free” of the hospital.

Primary Analysis Population

The primary analysis population, used for all adaptive analyses is the “As-Infused” population. The intent-to-treat population includes all patients randomized to an mAB arm. The “as-infused” population includes those patients that show up and are infused for their mAB. Given that all arms are a mAB arm, there is no anticipated relationship between lack of infusion and the assigned arm. Hence all adaptive analyses and safety analyses will be based on the as-infused population (where patients are coded by the mAB arm they receive). Patients who receive a randomized mAB allocation and are not infused may be tracked as a real-world evidence arm of outcome for a non-mAB control (see secondary analyses).

Primary Analysis Model

The primary analysis model for the primary endpoint is a cumulative proportional odds model. Let the probability of an outcome of less than or equal to y be $\pi_y = Pr(Y \leq y)$. Let *a* be the indicator of treatment arm (*a*=1,…,k). The model adjusts for the following baseline variables:

1. ED or infusion center (0=infusion center, 1=ED)
2. Age (with categories of <30, 30-39, 40-49, 50-59, 60-69, 70-79, and ≥80; 60-69 will be used as the referent)
3. Sex (sex at birth, male is the referent)
4. COVID-19 variant (0=unknown/uncollected (referent), categorical endpoint for each known variant)
5. Time (two-week epochs of time are used for adjustments; the most current 4-week period is the referent)

The primary analysis model is based on a cumulative logistic regression, where $\pi_y = \Pr(Y \leq y)$, where

$$\log\left(\frac{\pi_y}{1-\pi_y}\right) = \alpha_y - \theta_a \delta_{[a]} - \sum_{v=1}^{4} \beta_{vj}\delta_{vj} - \lambda_{[time]};\ y = -1,0,1,2,\ldots 27.$$

The additive covariate effects across all treatment arms for each patient are modeled through the β parameters. The δ parameters are indicator functions for the treatment arm and covariate values for the baseline covariates. The efficacy of the treatment arms is modeled with the θ parameters. The ordinal effect parameters (α_y) are modeled with a Dirichlet distribution with equal weight on each outcome and a sum of 1.

The baseline covariate effects are modeled with independent weak prior distributions:

$$\beta_{vj} \sim N(0,10^2), v = 1,\ldots,4;\ j = 1,\ldots,n_v.$$

The appropriate coefficients will be set to 0 within each covariate for identifiability (the goal will be the largest category set to 0).

The effects of time are adjusted within the model using two-week epochs and a smoothing model over time. The modeling of the time effects is set up with the most current period (2 epochs combined being the most recent month are set to 0):

$$\lambda_1 \equiv \lambda_2 \equiv 0$$

$$\lambda_2 - \lambda_1 \sim N(0, 0.15^2)$$

$$\lambda_T - 2\lambda_{T-1} + \lambda_{T-2} \sim N\left(0, \tau_\lambda^2\right); T \geq 3$$

$$\tau_\lambda^2 \sim IG(0.25, 0.00562)$$

The prior distributions for the mAB treatment effects are weak:

$$\theta_a \sim N(0,10), a = 1, \ldots, k.$$

Arms

One of the treatment arms is selected as the referent arm for treatment effects and assigned a treatment effect of $\boldsymbol{\theta_a = 0}$. The treatment arm at the first adaptive analysis with the largest sample size will be specified as the referent arm for the remainder of the trial.

Model Convergence

Given the complexity of the model, conventions may be taken by the analysis team if there are convergence issues or model stability issues. For example, there may be outcome categories in the 30 possible primary outcome values (e.g., k number of hospital-free days, patient death) that do not occur. If this happens at analysis, the cells will be combined to achieve model convergence. For example, if the 4 hospital-free day outcome value does not occur it will be combined with 3, and so on, until every cell has occurred. Additional model stability conventions will be taken to preserve the model stability.

Missing and Partial Data

If there are missing covariates for a patient in the as-infused patient population, the following conventions will be used.

1. If the treatment arm is missing the patient will be ignored.
2. If a baseline covariate is missing the referent value for that covariate will be used

For all model analyses, only patients who have achieved 28-days of follow-up from the date of the index infusion will be used in the analysis. No use or imputation of patient data for patients with less than 28 days will be conducted.

Given the EHR-based data summaries there will be no missing outcome data. If there is deemed to be a corrupted outcome that patient will be ignored. Some patients may have 28 hospitalization-free days that at subsequent analyses are found to have out of system hospitalizations. The data will be updated at future analyses.

Trial Inferences

For the primary analysis, there is no "control" treatment and so all inferences are made comparing the individual treatment arms to each other. The main quantity of interest will be the relative odds ratio between any two treatments arms

$$OR_{ij} = \exp(\theta_i)/\exp(\theta_j).$$

The posterior probability that the odds ratio for arm *i* compared to arm *j* is greater than 1 (signifying that treatment *i* is superior to treatment *j*) is used as a comparison between arms. Additionally, the posterior mean and 95% confidence interval between arms will be used to summarize relative treatment effects.

Adaptive Design

The trial design is adaptive. A sequence of frequent interim analyses will be conducted as a function of enrollment rate. The expectation is to conduct monthly adaptive analyses. The following decision triggers will be addressed at each adaptive analysis:

Arm Inferiority: If one of the arms has a 99% chance of being inferior to any of the other available arms then the inferior arm will be declared inferior and may be removed from the trial. There may be conditions of the pandemic (e.g., variant frequency, new variants) or drug supply concerns that an arm is retained.

Equivalence: Any two arms in the trial may reach a declaration of equivalence. It is anticipated that no actions would take when equivalence is reached but a declaration and public disclosure may be made. There is a sliding scale of equivalence with different levels of equivalence bounds. A declaration of equivalence will be tied to the equivalence level. Equivalence with a bound of d is declared if the posterior probability of the odds ratio is with d is at least 95%:

$$\Pr\left(\frac{1}{1+\delta} \leq OR_{ij} \leq 1+\delta\right) \geq 0.95$$

The following levels are pre-defined:

1. The first level of equivalence occurs when there is 95% posterior probability that the odds-ratio is within a bound of d=0.25
2. The second level of equivalence occurs when there is a 95% posterior probability that the odds-ratio is within a bound of d=0.20
3. The third level of equivalence occurs when there is a 95% posterior probability that the odds-ratio is within a bound of d=0.15
4. The fourth level of equivalence occurs when there is a 95% posterior probability that the odds-ratio is within a bound of d=0.10
5. The fifth level of equivalence occurs when there is a 95% posterior probability that the odds-ratio is within a bound of d=0.05

Combination Futility: When combinations of mAB are used the combination therapy may be compared to individual components of the combination. If there is more than a 95% probability that the effect of the combination mAB is no better than a 20% improvement in the odds ratio compared to each individual component then the combination may be declared *not clinically relevantly superior (futile)* to the individual components and the combination will be stopped. The comparison between a combination and the individual components will be declared when the combination is included within the platform trial.

Trial Read-Outs: There may be periodic "unblinding" of the trial results. When an arm is removed for inferiority or futility, the results for the inferior and the superior or the combination and its components of the two arms in the trigger will be unblinded and publicly released. The trial will continue and will utilize all data for any new inferences in the trial. Additionally, there may be need for periodic disclosure of the current trial results, such as when FDA revokes authorization for a given mAB. These disclosures will be made and the trial will continue unchanged.

Response-Adaptive Randomization: Assuming R arms available in the trial, response adaptive randomization will be utilized. The response adaptive randomization is conducted based on the probability that each arm has the optimal treatment effect (largest θ). Let q_a be the posterior probability that arm a is the optimal arm among the R arms in the randomization arm space:

$$q_a = \Pr(\theta_a = \max\{\theta_1, \ldots, \theta_R\})$$

The randomization probabilities are weighted toward being equal to maintain sufficient randomization to each arm and because the assignment is open-label, to prevent any obvious patterns of assignments. The allocation probability for each arm is

$$\text{Allocation probability} = \frac{q_a + 1/R}{1+1}.$$

If an arm joins the trial and has no data on the primary endpoint, then the value of q_a for that arm is assigned to be 1/R and the remaining arms probability of optimal summing to $1 - \frac{1}{R}$. This convention will create fixed randomization to a new arm in the trial until there is at least 1 observation of the primary endpoint for modeling.

Modeling Treatment Heterogeneity

During the course of the trial, and during a trial read-out, inferences of relative treatment effect by different subgroups may be utilized. In these cases, the treatment effect is modeled as a function of subgroup within the single larger model. Each level of the subgroup will be identified and added as a covariate in the model and then the treatment effect, θ_a, will be modeled separately within each subgroup, $s = 1, \ldots, S$, as $\theta_{a1}, \theta_{a2}, \ldots, \theta_{aS}$, with hierarchical prior distribution:

$$\theta_{as} \sim N(\mu_a, {\tau_a}^2), a = 1, \ldots, k;\ s = 1, \ldots, S.$$

$$\mu_a \sim N(0,10^2); \tau_a^2 \sim IG(0.25, 0.1).$$

Assessment of COVID-19 Variants

The COVID-19 variant type is a predefined subgroup for analysis. The variant type for each patient is unknown at baseline, but samples will be collected and reported upon sequencing. The posterior median odds ratios and 95% credible intervals will be reported for each arm comparison within each variant subset. In addition, because a large percentage of mAB infused patients will not have variant sequencing data (at least in near real time), a second "surrogate" approach will be used to compare the relative efficacy of the mAB regimens in the full treated population over time and by variant type.

Using Pennsylvania statewide data, we will estimate the prevalence of a given variant type by time. Then, we can categorize patients into various time epochs relative to the variant prevalence. These categories will be provided based upon the changing distributions over time in our region.

To consider the internal validity of this “surrogate” approach, we will use data from the subset of patients with actual genotype sequencing, compare the proportion of patients with prevalence of the variant type of interest across levels of the surrogate classification (i.e., Cochran Mantel-Haenszel test of trend).

Adaptive Analyses Reporting

The primary analysis will read out with summaries of the potential arm triggers for each arm actively in the “regimen space.” These summaries will include the probability each mAB is optimal in the active regimen space and the randomization probabilities.

A second analysis will be conducted with all arms (even those closed) in the regimen space, to report the probability each regimen is optimal among the larger regimen space.

As the EHR system contains the same covariate and baseline data for both mAB treated and untreated patients, we have continuously updated flags for each patient in the system as being “mAB eligible” (or not) based on EUA criteria on the date the patient became COVID-19 positive. With appropriate selection, this affords 2 untreated control groups for analysis that we will describe using mean (SD), median [IQR}, and proportions, as appropriate: mAB eligible patients who were never randomly assigned to a respective mAB regimen, and mAB eligible patients who were randomly assigned to a mAB regimen yet did not receive treatment.

SAP Read-Out for October 28, 2021

Date created: November 4, 2021

Introduction

This document describes the detail of the analysis read-out for the October 28, 2021 dataset. This document is an appendix to the Statistical Analysis Plan for the "UPMC Antibody Treatment and Evaluation Center", and is a follow-up to the read-out for September 30, 2021, now that the trial population of interest (July 14 to September 29, 2021) has passed 28 day follow-up.

The September 30, 2021 read-out was conducted early, prior to all patients having reached 28 day follow-up, due to the Delta variant crisis.

- We will analyze and report two treatment arms (below).

- Unblinded data from this analysis can be shared with investigators, to prepare the public report. This unblinding is appropriate as there are no "control" arms as all patients receive mAB treatment, and the urgency of Delta.

    - Results from the primary analysis can be released first to investigators before sensitivity and subgroup analyses are complete.

- UPMC continues to provide treatment with all available mAbs, with randomization allocation.

Treatment Arms
There are two treatment arms that will be included in this analysis. The arms are:

1. C+I (casirivimab and imdevimab)
2. S (sotrovimab)

B+E (bamlanivimab and etesevimab) became available again at UPMC on September 16, 2021, due to a U.S. Government decision. However, only a small amount of patients received B+E from September 16 to September 29, 2021, relative to the much larger number that received C+I or S for the trial period of interest. Therefore, we have little belief a strong statistical conclusion for B+E exists. In addition, we continue to randomize into B+E. For these reasons, we will not analyze B+E in this readout.

Primary Endpoint
The primary endpoint for this read-out is hospital-free days at day 28.

Primary Analysis Population
The primary analysis population for this read-out is the "As-Infused" population. This analysis will include all patients infused with a randomized mAb allocation in an ED or infusion center from July 14, 2021 until September 29, 2021. The date of the data set snapshot is on October 28, 2021. This analysis will not include any patients infused before July 14, 2021.

Due to mAb shortages, the below two situations arose and will be analyzed as follows:

1. No therapeutic interchange possible (InterChangeIndic)

Due to episodic mAb shortages, some patients were treated at sites with only 1 available mAb at the time of treatment. However, prescribing physicians and patients were unaware of drug availability at time of randomization, a patient could be randomized to a different mAb on the same day and city (just with different availability by site), and bias is unlikely in the patient types or clinical management during the times when only 1 mAb was available at a given site.

Therefore, these patients will be included in the primary analysis. This variable will be included in the dataset for this readout and will be used in a sensitivity analysis where these patients will be excluded (page 4).

2. Not IV route (SUBQ_FLAG)

To increase treatment capacity, UPMC began SQ (subcutaneous) C+I injections starting in mid-September as more SQ treatments can be done per day than IV. We sought to compare mAbs given via the same route (IV) and the primary analysis population is the "As-Infused" population.

Therefore, these patients will be excluded from the primary analysis. This variable will be included in the dataset for this readout, for only descriptive purposes.

Primary Analysis Model

The primary analysis model is as described in the trial SAP.

The primary analysis for the primary endpoint is a cumulative proportional odds model. Let the probability of an outcome of less than or equal to y be $\pi_y = Pr(Y \le y)$. Let *a* be the indicator of treatment arm (*a*=1,…,k). The model adjusts for the following baseline variables:

6. ED or infusion center (0=infusion center, 1=ED)
7. Age (with categories of <30, 30-39, 40-49, 50-59, 60-69, 70-79, and ≥80; 60-69 will be used as the referent)
8. Sex (sex at birth, male is the referent)
~~9.~~ Covid variant is not modeled in this primary analysis, as the vast majority of all variants measured at UPMC and in state-wide GSAID data have been Delta for this readout's time period.
10. Time (two-week epochs of time are used for adjustments; the most current 4-week period is the referent)

The primary analysis model is based on a cumulative logistic regression, where $\pi_y = \Pr(Y \le y)$, where

$$\log\left(\frac{\pi_y}{1-\pi_y}\right) = \alpha_y - \theta_a\delta_{[a]} - \sum_{v=1}^{4}\beta_{vj}\delta_{vj} - \lambda_{[time]};\ y = -1,0,1,2,\dots 27.$$

The additive covariate effects across all treatment arms for each patient are modeled through the β parameters. The δ parameters are indicator functions for the treatment arm and covariate values for the baseline covariates. The efficacy of the treatment arms is modeled with the θ parameters. The

ordinal effect parameters (α_y) are modeled with a Dirichlet distribution with equal weight on each outcome and a sum of 1.

The baseline covariate effects are modeled with independent weak prior distributions:

$$\beta_{vj} \sim N(0,10^2), v = 1, \ldots ,4;\ j = 1, \ldots , n_v.$$

The appropriate coefficients will be set to 0 within each covariate for identifiability (the goal will be the largest category set to 0).

The effects of time are adjusted within the model using two-week epochs and a smoothing model over time. The modeling of the time effects is set up with the most current period (2 epochs combined being the most recent month are set to 0):

$$\lambda_1 \equiv \lambda_2 \equiv 0$$

$$\lambda_2 - \lambda_1 \sim N(0, 0.15^2)$$

$$\lambda_T - 2\lambda_{T-1} + \lambda_{T-2} \sim N\left(0, \tau_\lambda^2\right); T \geq 3$$

$$\tau_\lambda^2 \sim IG(0.25, 0.00562)$$

The prior distributions for the mAB treatment effects are weak:

$$\theta_a \sim N(0,10), a = 1,2.$$

The treatment arm with the largest sample size should be selected as the referent arm (label a=1)_for treatment effects and assigned a treatment effect of $\theta_1 = 0$.

Model Convergence

Given the complexity of the model, conventions may be taken by the analysis team if there are convergence issues or model stability issues. For example, there may be outcome categories in the 30 possible primary outcome values (e.g., k number of hospital-free days, patient death) that do not occur. If this happens at analysis, the cells will be combined to achieve model convergence. For example, if the 4 hospital-free day outcome value does not occur it will be combined with 3, and so on, until every cell has occurred. Additional model stability conventions will be taken to preserve the model stability.

Missing and Partial Data

If there are missing covariates for a patient in the as-infused patient population, the following conventions will be used.

3. If the treatment arm is missing the patient will be ignored.
4. If a baseline covariate is missing the referent value for that covariate will be used

For all model analyses, only patients who have achieved 28-days of follow-up from the date of the index infusion will be used in the primary analysis. No use or imputation of patient data for patients with less than 28 days will be conducted.

Given the EHR-based data summaries there will be no missing outcome data, barring patients who received treatment less than 28 days ago. If there is deemed to be a corrupted outcome that patient will

be ignored. Some patients may have 28 hospitalization-free days that at subsequent analyses are found to have out of system hospitalizations. The data will be updated at future analyses.

Sensitivity Analyses
We will conduct the following sensitivity analysis:

- Exclude the no therapeutic interchange possible patients (InterChangeIndic)

Subgroup Analyses

For subgroup analyses the model is fit independently in each subgroup classification. If the subgroup is a covariate in the model that covariate is removed from the analysis.

1. A priori, per approved protocol

- By vaccine status (fully, partially, unvaccinated, unknown)

2. Post hoc subgroup analyses

- By symptom onset (>5 days or $\leq$5 days)
- By location (0=infusion center, 1=ED)

Trial Inferences
For the primary analysis, there is no "control" treatment and so all inferences are made comparing the individual treatment arms to each other. The main quantity of interest will be the relative odds ratio between any two treatments arms

$$OR_{ij} = \exp(\theta_i)/\exp(\theta_j).$$

The posterior probability that the odds ratio for arm *i* compared to arm *j* is greater than 1 (signifying that treatment *i* is superior to treatment *j*) is used as a comparison between arms. Additionally, the posterior mean and 95% confidence interval between arms will be used to summarize relative treatment effects.

Arm Inferiority: If one of the arms has a 99% chance of being inferior to any of the other available arms then the inferior arm will be declared inferior and may be removed from the trial. There may be conditions of the pandemic (variation frequency, new variations) or drug supply concerns that an arm is retained.

Equivalence: Any two arms in the trial may reach a declaration of equivalence. It is anticipated that no actions would take when equivalence is reached but a declaration and public disclosure may be made. There is a sliding scale of equivalence with different levels of equivalence bounds. A declaration of equivalence will be tied to the equivalence level. Equivalence with a bound of d is declared if the posterior probability of the odds ratio is with d is at least 95%:

$$\Pr\left(\frac{1}{1+\delta} \leq OR_{ij} \leq 1+\delta\right) \geq 0.95$$

The following levels are pre-defined:

6. The first level of equivalence occurs when there is 95% posterior probability that the odds-ratio is within a bound of d=0.25
7. The second level of equivalence occurs when there is a 95% posterior probability that the odds-ratio is within a bound of d=0.20
8. The third level of equivalence occurs when there is a 95% posterior probability that the odds-ratio is within a bound of d=0.15
9. The fourth level of equivalence occurs when there is a 95% posterior probability that the odds-ratio is within a bound of d=0.10
10. The fifth level of equivalence occurs when there is a 95% posterior probability that the odds-ratio is within a bound of d=0.05

Dataset

- Pitt Data Freeze on 10/28/2021
- 2 treatment arms for primary analysis
  - C+I (casirivimab-imdevimab)
  - S (sotrovimab)
- Include patients <u>infused</u> from 0:00a July 14, 2021 until September 29, 11:59p
  - No patients from before July 14, 2021
- No patients who received treatment less than 28 days ago
- Included for descriptive purposes (not for primary analysis)
  - Patients who received SQ (subcutaneous) injections
  - Patients who received B+E (bamlanivimab and etesevimab)
- **InterChangeIndic**
- **Vaccination Status**
- **Symptom Onset Date**
- **SUBQ_FLAG**
- **ReasonNotInfused**
  - Admitted
  - Declined
  - Ineligible
  - Infusion Incomplete
  - Pending return call
  - Scheduled
  - Unable to contact
  - Unable to travel

Specific Analyses

#	Status	Population	Endpoint	Other
**1**	Primary	As-Infused to C+I or S from July 14, 2021 until September 29, 2021	HFD	Sensitivity and subgroup analyses will also be conducted as detailed above.

1. Primary Analysis

The following posterior probabilities will be reported:

Quantity of Interest	Posterior Probability
C+I inferior to S	
S equivalent to C+I (as per the pre-defined 5 levels and associated bounds)	
S inferior to C+I	

Statistical Triggers Met for Equivalence with delta ranges of equivalence

The following will be reported:

Odds-Ratio Parameter	Mean	SD	Median	95% Credible Interval
Age < 30				
Age 30 – 39				
Age 40 – 49				
Age 50 – 59				
Age 60 – 69	1			
Age 70 – 79				
Age 80+				
Female				
Time Bucket 1				
…				
Time Bucket k-1				
Referent Arm	1			
Arm #2				

Graphical summaries:

1. Stacked bar plots and cumulative distributions of HFDs by treatment arm
2. Stacked bar plots and cumulative distributions of HFDs by location
3. Stacked bar plots and cumulative distributions of HFDs by vaccine status
4. Stacked bar plots and cumulative distributions of HFDs by symptom onset

Sample size estimates

Separate from this readout, a blinded statistician will generate sample size estimates for reaching the pre-specified statistical thresholds for inferiority and equivalence (at the 5 pre-specified levels) between C+I and S, based on the results seen in this report.
