# Supplementary material for: Effectiveness of Casirivimab-Imdevimab and Sotrovimab During a SARS-CoV-2 Delta Variant Surge: A Cohort Study and Randomized Comparative Effectiveness Trial
Source: JAMA Netw Open. 2022 Jul 14;5(7):e2220957. doi: 10.1001/jamanetworkopen.2022.20957 (PMC10881222; doi:10.1001/jamanetworkopen.2022.20957)
Supplement: Supplement 2. — eFigure 1. SARS-CoV-2 Variants of Concern Proportion in Pennsylvania During the Study eFigure 2. Propensity Scores for Unmatched and Matched Patients eTable 1. Comparison of Characteristics of Unmatched and Propensity Matched Patients eTable 2. Primary and Secondary Outcomes in an Unmatched Cohort of Patients Receiving Monoclonal Antibody Treatment and an At-Risk Population of Patients Not Receiving Monoclonal Antibody Treatment eTable 3. Subgroup Analyses of the Randomized Comparative Effectiveness Trial, Comparing Sotrovimab to Casirivimab and Imdevimab [file jamanetwopen-e2220957-s002.pdf]

Supplemental Online Content

Huang DT, McCreary EK, Bariola JR, et al. Effectiveness of casirivimab-imdevimab and sotrovimab during a SARS-CoV-2 Delta variant surge: a cohort study and randomized comparative effectiveness trial. *JAMA Netw Open*. 2022;5(7):e2220957. doi:10.1001/jamanetworkopen.2022.20957

eFigure 1. SARS-CoV-2 Variants of Concern Proportion in Pennsylvania During the Study

eFigure 2. Propensity Scores for Unmatched and Matched Patients

eTable 1. Comparison of Characteristics of Unmatched and Propensity Matched Patients

eTable 2. Primary and Secondary Outcomes in an Unmatched Cohort of Patients Receiving Monoclonal Antibody Treatment and an At-Risk Population of Patients Not Receiving Monoclonal Antibody Treatment

eTable 3. Subgroup Analyses of the Randomized Comparative Effectiveness Trial, Comparing Sotrovimab to Casirivimab and Imdevimab

This supplemental material has been provided by the authors to give readers additional information about their work.

eFigure 1. SARS-CoV-2 Variants of Concern Proportion in Pennsylvania During the Study

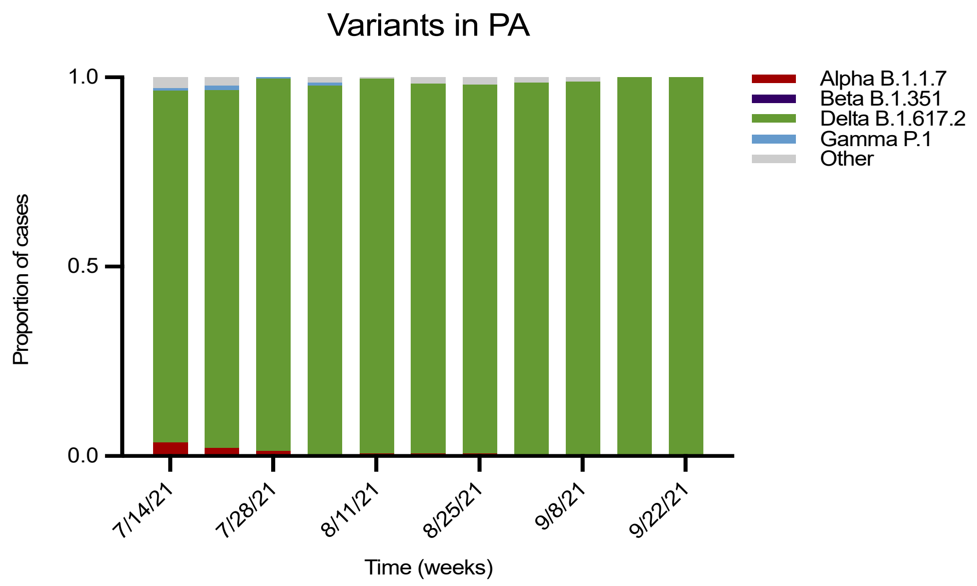

Red represents Alpha B.1.1.7, purple is Beta B.1.351, green is Delta B.1.617.2, and blue is Gamma P.1. There were no B.1.351 cases in PA during this time frame.

© 2022 Huang DT et al. *JAMA Network Open.*

eFigure 2. Propensity Scores for Unmatched and Matched Patients

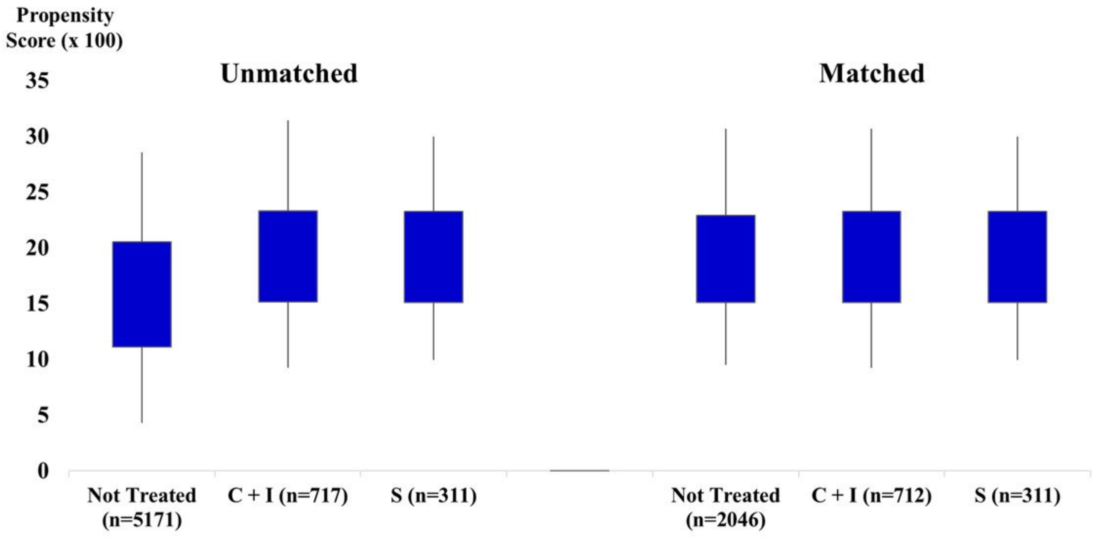

After propensity score matching, treated and non-mAb treated patients were similar on the distribution of propensity scores.

Abbreviations: C + I - Casirivimab + Imdevimab; S - Sotrovimab

© 2022 Huang DT et al. *JAMA Network Open.*

eTable 1. Comparison of Characteristics of Unmatched and Propensity Matched Patients

	Unmatched			Propensity Matched		
	Treated	Not Treated	p-value	Treated	Not Treated	p-value
No.	1,028	5,171		1,023	2,046	
Patient Characteristics						
Age, mean (SD)	53.2 (16.4)	49.6 (21.4)	<.001	53.2 (16.4)	52.8 (19.5)	.50
Female gender, (No.), %	(572) 55.6%	(3013) 58.3%	.12	(569) 55.6%	(1157) 56.6%	.62
Black race, (No.), %	(61) 5.9%	(590) 11.4%	<.001	(61) 6.0%	(112) 5.5%	.58
Body mass index, mean (SD)	32.5 (7.4)	31.8 (7.5)	.008	32.5 (7.4)	32.6 (7.8)	.60
Medical History						
History of cancer, (No.), %	(46) 4.5%	(597) 11.6%	<.001	(42) 4.1%	(105) 5.1%	.21
History of congestive heart failure, (No.), %	(47) 4.6%	(399) 7.7%	<.001	(46) 4.5%	(93) 4.6%	.95
History of obstructive sleep apnea, (No.), %	(198) 19.3%	(743) 14.4%	<.001	(195) 19.1%	(338) 16.5%	.08
Charlson Comorbidity Index Score, mean (SD)	0.7 (0.9)	0.9 (1.2)	<.001	0.7 (0.9)	0.7 (0.9)	.57
History of rheumatoid arthritis, (No.), %	(38) 3.7%	(104) 2.0%	.001	(34) 3.3%	(61) 3.0%	.61
History of atrial fibrillation, (No.), %	(46) 4.5%	(307) 5.9%	.06	(46) 4.5%	(90) 4.4%	.90
History of valvular heart disease, (No.), %	(73) 7.1%	(287) 5.6%	.05	(72) 7.0%	(138) 6.7%	.76
History of viral hepatitis, (No.), %	(7) 0.7%	(79) 1.5%	.03	(7) 0.7%	(16) 0.8%	.77
Antidepressants, (No.), %	(335) 32.6%	(1497) 29.0%	.02	(330) 32.3%	(674) 32.9%	.70
History of asthma, (No.), %	(312) 30.4%	(1851) 35.8%	<.001	(311) 30.4%	(593) 29.0%	.42
History of stroke, (No.), %	(45) 4.4%	(305) 5.9%	.05	(45) 4.4%	(91) 4.5%	.95
History of allergic rhinitis, (No.), %	(146) 14.2%	(658) 12.7%	.20	(145) 14.2%	(268) 13.1%	.41
Alpha blockers, (No.), %	(11) 1.1%	(78) 1.5%	.28	(11) 1.1%	(20) 1.0%	.80
Variables Not Included in Propensity Model						
ACE Inhibitors, (No.), %	(144) 14.0%	(759) 14.7%	.58	(144) 14.1%	(303) 14.8%	.59
Angiotensin II receptor blocker, (No.), %	(104) 10.1%	(450) 8.7%	.15	(102) 10.0%	(195) 9.5%	.70
Beta blockers, (No.), %	(215) 20.9%	(1114) 21.5%	.65	(212) 20.7%	(462) 22.6%	.24
History of coronary artery disease, (No.), %	(104) 10.1%	(542) 10.5%	.73	(103) 10.1%	(188) 9.2%	.43
History of chronic kidney disease, (No.), %	(53) 5.2%	(336) 6.5%	.11	(53) 5.2%	(117) 5.7%	.54
History of COPD, (No.), %	(148) 14.4%	(831) 16.1%	.18	(146) 14.3%	(272) 13.3%	.46
Corticosteroids, (No.), %	(348) 33.9%	(1639) 31.7%	.18	(345) 33.7%	(653) 31.9%	.31
History of diabetes, (No.), %	(167) 16.3%	(883) 17.1%	.52	(166) 16.2%	(331) 16.2%	.97
History of dyspnea, (No.), %	(51) 5.0%	(295) 5.7%	.34	(51) 5.0%	(104) 5.1%	.91
History of fatty liver disease, (No.), %	(34) 3.3%	(143) 2.8%	.34	(34) 3.3%	(59) 2.9%	.50
History of hypertension, (No.), %	(426) 41.4%	(2070) 40.0%	.40	(423) 41.4%	(861) 42.1%	.70
History of pulmonary hypertension, (No.), %	(8) 0.8%	(102) 2.0%	.008	(8) 0.8%	(30) 1.5%	.11

© 2022 Huang DT et al. *JAMA Network Open.*

Statins, (No.), %	(320) 31.1%	(1485) 28.7%	.12	(318) 31.1%	(630) 30.8%	.87
Abbreviations: COPD - chronic obstructive pulmonary disease						

© 2022 Huang DT et al. *JAMA Network Open.*

eTable 2. Primary and Secondary Outcomes in an Unmatched Cohort of Patients Receiving Monoclonal Antibody Treatment and an At-Risk Population of Patients Not Receiving Monoclonal Antibody Treatment

	(No. Events), Rate %		Risk Ratio (RR) Estimates						
			Unadj.	Adjusted by Propensity Score			Adjusted by IPW		
	Treated	Not Treated	RR	RR	(95% CI)	p-value	RR	(95% CI)	p-value
Patient Outcomes									
Casirivimab + Imdevimab vs. Not Treated									
No.	717	5,171							
Hospitalization or mortality	(19) 2.6%	(479) 9.3%	0.29	0.27	(0.17–0.42)	<.001	0.41	(0.34–0.48)	<.001
Hospitalization	(19) 2.6%	(357) 6.9%	0.38	0.36	(0.23–0.57)	<.001	0.54	(0.46–0.65)	<.001
Mortality	(1) 0.1%	(184) 3.6%	0.04	0.04	(0.01–0.26)	.001	0.02	(0.01–0.06)	<.001
Sotrovimab vs. Not Treated									
No.	311	5,171							
Hospitalization or mortality	(16) 5.1%	(479) 9.3%	0.75	0.72	(0.57–0.92)	.009	0.76	(0.69–0.85)	<.001
Hospitalization	(16) 5.1%	(357) 6.9%	0.86	0.84	(0.66–1.07)	.16	0.88	(0.80–0.98)	.02
Mortality	(0) 0.0%	(184) 3.6%	0.00	---	---	---	---	---	---
All mAb vs. Not Treated									
No.	1,028	5,171							
Hospitalization or mortality	(35) 3.4%	(479) 9.3%	0.37	0.35	(0.25–0.49)	<.001	0.46	(0.40–0.53)	<.001
Hospitalization	(35) 3.4%	(357) 6.9%	0.49	0.47	(0.33–0.66)	<.001	0.62	(0.53–0.71)	<.001
Mortality	(1) 0.1%	(184) 3.6%	0.03	0.03	(0.01–0.18)	<.001	0.01	(0.01–0.04)	<.001

Abbreviations: Unadj - unadjusted; CI - confidence interval; IPW - inverse probability weight; mAb - monoclonal antibodies

© 2022 Huang DT et al. *JAMA Network Open.*

eTable 3. Subgroup Analyses of the Randomized Comparative Effectiveness Trial, Comparing Sotrovimab to Casirivimab and Imdevimab

	N	Odds ratio, sotrovimab vs casirivimab and imdevimab; median (95% credible interval)	Probability of inferiority of sotrovimab vs casirivimab and imdevimab; %
Vaccine status			
Full	934	0.76 (0.44 – 1.32)	83.3
Partial	121	0.27 (0.08 – 0.88)	98.5
Unvaccinated	683	0.75 (0.36 – 1.63)	77.4
Unknown	1820	1.04 (0.78 – 1.39)	40.1
Symptom onset			
>5 days	970	0.72 (0.36 – 1.47)	82.0
≤5 days	657	1.04 (0.49 - 2.33)	46.2
Unknown	1931	-	-
Location			
Infusion center	1511	0.48 (0.28 – 0.84)	99.5
Emergency department	2047	0.99 (0.76 – 1.28)	53.7

© 2022 Huang DT et al. *JAMA Network Open.*
