# Supplementary material for: Effectiveness of Casirivimab-Imdevimab and Sotrovimab During a SARS-CoV-2 Delta Variant Surge: A Cohort Study and Randomized Comparative Effectiveness Trial
Source: JAMA Netw Open. 2022 Jul 14;5(7):e2220957. doi: 10.1001/jamanetworkopen.2022.20957 (PMC10881222; doi:10.1001/jamanetworkopen.2022.20957)
Supplement: Supplement 3. — Data Sharing Statement [file jamanetwopen-e2220957-s003.pdf]

# Data Sharing Statement

Huang. Effectiveness of Casirivimab-Imdevimab and Sotrovimab During a SARS-CoV-2 Delta Variant Surge. *JAMA Netw Open*. Published July 14, 2022.
doi:10.1001/jamanetworkopen.2022.20957

**Data**
**Data available:** No
